# Evaluation of Cell Mimics as Potential Quality Controls for Human Leukocyte Immunophenotyping

**DOI:** 10.3390/medsci13040314

**Published:** 2025-12-11

**Authors:** Louis Waeckel, Brigitte Le Mauff, Jacques Trauet, Julie Demaret, Ahmed Boumediene, Margarita Hurtado-Nedelec, Arnaud Ciree, Gwladys Bourdenet, Claude Lambert

**Affiliations:** 1 Laboratoire d’Immunologie, CHU Saint-Etienne, 42055 Saint-Etienne Cedex 2, France; louis.waeckel@chu-st-etienne.fr; 2 Laboratoire d’Immunologie, CHU Caen, 14000 Caen, France; lemauff-b@chu-caen.fr; 3 Institut d’Immunologie Laboratoire de Déficits Immunitaires et Immunothérapies, CHRU de Lille, F-59000 Lille, France; jacques.trauet@chu-lille.fr (J.T.); julie.demaret@chu-lille.fr (J.D.); 4 Laboratoire d’Immunologie et d’Immunogénétique, CHU Limoges, 87042 Limoges, France; ahmed.boumediene@chu-limoges.fr; 5 Laboratoire d’Hématologie et Immunologie, APHP, Hôpital Bichat, 75018 Paris, France; maria.hurtado-nedelec@aphp.fr; 6 Laboratoire d’Immunologie, CHRU Bretonneau Tours, 37044 Tours, France; a.ciree@chu-tours.fr; 7 Laboratoire d’Immunologie, CHU Amiens Picardie, 80054 Amiens, France; bourdenet.gwladys@chu-amiens.fr

**Keywords:** flow cytometry, quality controls, cell substitutes, artificial cell mimics, FlowCytes, TruCytes, absolute count, quality assurance

## Abstract

**Background**. Routine lymphocyte counting requires quality assurance validation using quality controls (QCs) that closely resemble fresh human blood leukocytes. This study aimed to evaluate a novel artificial product designed to mimic leukocyte light scatters and marker expression. **Methods**. FlowCytes and TruCytes, “artificial cell mimics” (Slingshot Biosciences, Emeryville, CA, USA), were tested on CE-IVD-certified systems, namely FACSCanto, FACSLyric (BD Biosciences), Navios, and DxFlex (Beckman Coulter), using routine staining, lysing, fixation, and no-wash procedures for T, B, and NK counting. **Results**. FlowCytes and TruCytes provided forward and side scatter profiles comparable to human leukocytes on the FACSCanto, FACSLyric, and DxFlex systems but not on Navios despite adapting the process to be slightly different from the manufacturer’s recommendations. TruCytes demonstrated robust immunolabeling of CD3, CD4, CD8, and CD19 on the FACSCanto, FACSLyric, and DxFlex systems, with fluorescence intensities and subset distributions being similar to those usually observed in fresh human blood. However, CD16 and CD56 labeling was inconsistent and depended on the antibody clones used. Regrettably, monocyte and granulocyte mimics lacked expression of CD4, CD16, and CD14. TruCytes also displayed significantly lower concentrations of TBNK lymphocyte subsets compared to healthy human blood. **Conclusions**. FlowCytes and TruCytes show promises as internal quality controls for T cell and B cell immunophenotyping, but not NK cells. They are compatible with most CE-IVD cytometers, even when using lysis/fixation/no-wash routine diagnosis procedures. Further multicentric studies are warranted to assess their performance relative to existing products, such as stabilized human blood.

## 1. Introduction

According to quality assurance (QA) ISO15189 guidelines [1,2], proper identification of leukocyte subsets and reproducibility of cell quantitation must be verified daily using quality controls (QCs). For TBNK immunophenotyping, monoclonal antibodies targeting CD45, CD3, CD4, CD8, CD19, CD16, and CD56 are commonly used [3,4]. Several studies have demonstrated that in addition to the most conventional CD4+ and CD8+ T cell subsets, some T cells neither express CD4 nor CD8 (double negative), most of which are gamma-delta (gdT) T cells [5,6], while other T cells may co-express CD4 and CD8 at asymmetrical levels (CD4+ CD8dim or CD4dim CD8+ T cells [7,8]). Monocytes (CD14+, CD3−) also express CD4, albeit at lower levels than CD4+ T cells. CD16 is strongly expressed by neutrophils and at low levels by non-classical monocytes and part of NK cells [9]. CD56 is typically used to identify NK cells; however, due to its low expression, it is often used in conjunction with CD16, with anti-CD56 and anti-CD16 antibodies frequently conjugated to the same fluorochrome. NK cells must be distinguished from a small subset of T cells that express CD56 [9,10] and from non-classical monocytes that also express CD16 and are located in close proximity on FSC/SSC or CD45/SSC dot plots used to define the lymphocyte gate [11,12].

The precise determination of leukocyte subsets may vary due to the lack of consensus regarding panel design, instrument settings, staining protocols, and gating strategies [9]. Absolute counts are obtained using fluorescent beads as the internal standard, which introduces additional variability on the results compared to relative (%) counting. Best practice involves performing leukocyte analysis on fresh whole blood, followed by erythrolysis, fixation, and direct acquisition without washing (lysis/fixation/no-wash method). Acquisition is often performed at medium or high speed to save time because the no-wash procedure induces sample dilution. The most frequent lymphocyte counting systems in medical diagnosis include the FACSCanto or FACSLyric (both using FACSLysing solution and BD Trucount tubes from BD Biosciences) and Navios or DxFlex (using IMMUNOPREP lysing solution and Flow-Count beads from Beckman Coulter) [9].

To ensure reliable patient follow-up over time, laboratories must verify the reproducibility of the result on a daily basis using “internal QC (IQC)” to assess (1) labeling quality; (2) counting precision; and (3) counting reproducibility. In the absence of a gold standard, counting must at least be comparable to other laboratories, as evaluated by “external QC (EQC)” programs [2,13,14,15,16]. QC analysis should follow the same process as patient samples, i.e., “lyse, fix, no-wash”.

Currently, the most commonly used quality controls for TBNK immunophenotyping are stabilized human blood samples. However, these have limitations: they are not stable over time, and their manufacturing process can alter CD expression, complicating the gating of lymphocyte subsets.

Recently, synthetic products mimicking human leukocytes have been commercialized by Slingshot Biosciences (Inc. Emeryville, CA) as “artificial cell mimics” for use as QC in flow cytometry analyses: FlowCytes and TruCytes. FlowCytes are described as “synthetic white blood cells” intended as a forward and side scatter control for the identification and analysis of lymphocytes, monocytes, and granulocytes in whole blood. They are provided in suspension, ready to use, and can be shipped and stored at room temperature. TruCytes are cell mimics that, in addition, feature the following leukocyte surface markers according to the manufacturer: CD3 (using SK7 clone), CD4 (SK3), CD8 (SK1), CD16 (B73.1), CD19 (SJ25C1), CD45 (2D1), CD56 (NCAM16.2), and CD14. TruCytes are provided lyophilized, to be shipped and stored at −20 °C, in single test vials. The manufacturer recommends that upon reconstitution, TruCytes should be used within seven days if stored at 2–8 °C. Three washing steps are recommended but not mandatory. Data acquisition should be performed at a low flow rate. The manufacturer states that erythrolysis can be used safely on labeled TruCytes™. However, there are no specific instructions regarding the immunolabelling procedure (time and temperature) or fixation procedure. Neither FlowCytes nor TruCytes are IVD-certified; they are for Research Use Only (RUO).

The aim of this study was to evaluate whether FlowCytes and TruCytes could serve as quality controls for both relative and absolute TBNK counting on the most frequently used CE-IVD cytometers, using routine procedures that deviate from the manufacturer’s instructions.

## 2. Methods

### 2.1. Sample Selection

FlowCytes and TruCytes were provided by Slingshot Biosciences (Emeryville, CA, USA) and used according to each laboratory’s routine procedure (generally including lysis/fixation/no-wash) for whole blood. Due to the volume required for repeated analysis, samples were reconstituted in 500 µL distilled water (twice the recommended volume) and reproducibly stained either extemporaneously or after storage for up to seven days at 4–8 °C. Briefly, a TruCytes sample (50 to 100 µL depending to the local procedure) was incubated with the volume of antibodies usually used for the same volume of blood. Antibodies’ characteristics are detailed in Table 1. Samples were then incubated for 15–30 min followed by fixation and erythrolysis using either BD FACS Lysing Solution (BD Biosciences, San Jose, CA, USA) or IMMUNOPREP on TQ-Prep workstation (Beckman-Coulter, Fullerton, CA, USA). No washing step was performed, except in cases where the effects of washing were tested. Washing was then performed by adding 4 mL of PBS followed by centrifugation at 250× *g* for 10 min at room temperature. Samples were analyzed within 4 h, using either Navios (3 lasers and 10 colors), DxFLEX (3 lasers, 13 colors; Beckman-Coulter) or BD FACSLyric (3 lasers and 12 colors; BD Biosciences) cytometers, with the same instrument settings (PMT/ADP voltages, flow rates, and compensation) as those for routine blood immunophenotyping. Absolute counts were performed using BD Trucount tubes (BD Biosciences) or Flow-Count beads (Beckman-Coulter) according to the respective manufacturer’s instructions. Instrument settings were verified daily using CS&T beads (BD Biosciences), Flow-Check, Flow-Set, or DxFLEX DailyQC (Beckman-Coulter) according to respective manufacturer’s procedures.

Data were analyzed on the software usually routinely used for diagnosis in each accredited laboratory: Navios software (version 2.0), CytExpert (version 2.3.4.37), and BD FACSuite (version 1.6). Data were then independently re-analyzed using Kaluza software (Beckman-Coulter, version 2.3.1) to compare fluorescence intensities on the same scale. Sample quality was assessed using Side Scatter (SSC)/Forward Scatter (FSC) dot plots, and acquisition stability was verified on a time/SSC dot plot. Cell doublet exclusion using the FSC area/FCS height plotting was generally not performed to avoid the loss of calibration beads. Lymphocytes were gated based on CD45 labeling. NK cells and T cells were gated on the CD3/CD56-CD16 dot plot within the CD45+ lymphocyte populations. CD19+ B cells were gated on the CD3/CD19 dot plot within the CD45+ lymphocytes. CD4+ and CD8+ T cells were gated on a dot plot within the CD3+ T cells. In some laboratories, CD45/CD3/CD4/CD8 and CD45/CD3/CD19/CD56 stainings were performed in two separate tubes. Proportions (% of parent cells), absolute counts (cells/µL), and median fluorescence intensities (MdFI) were calculated.

### 2.2. Statistics

Results were expressed as mean +/− 1 standard deviation and compared using Student’s t-test. Figures, tables, and statistical analyses were performed using GraphPad (Prism). Statistical significance was set at *p* < 0.05.

## 3. Results

### 3.1. FlowCytes

**Figure 1:**
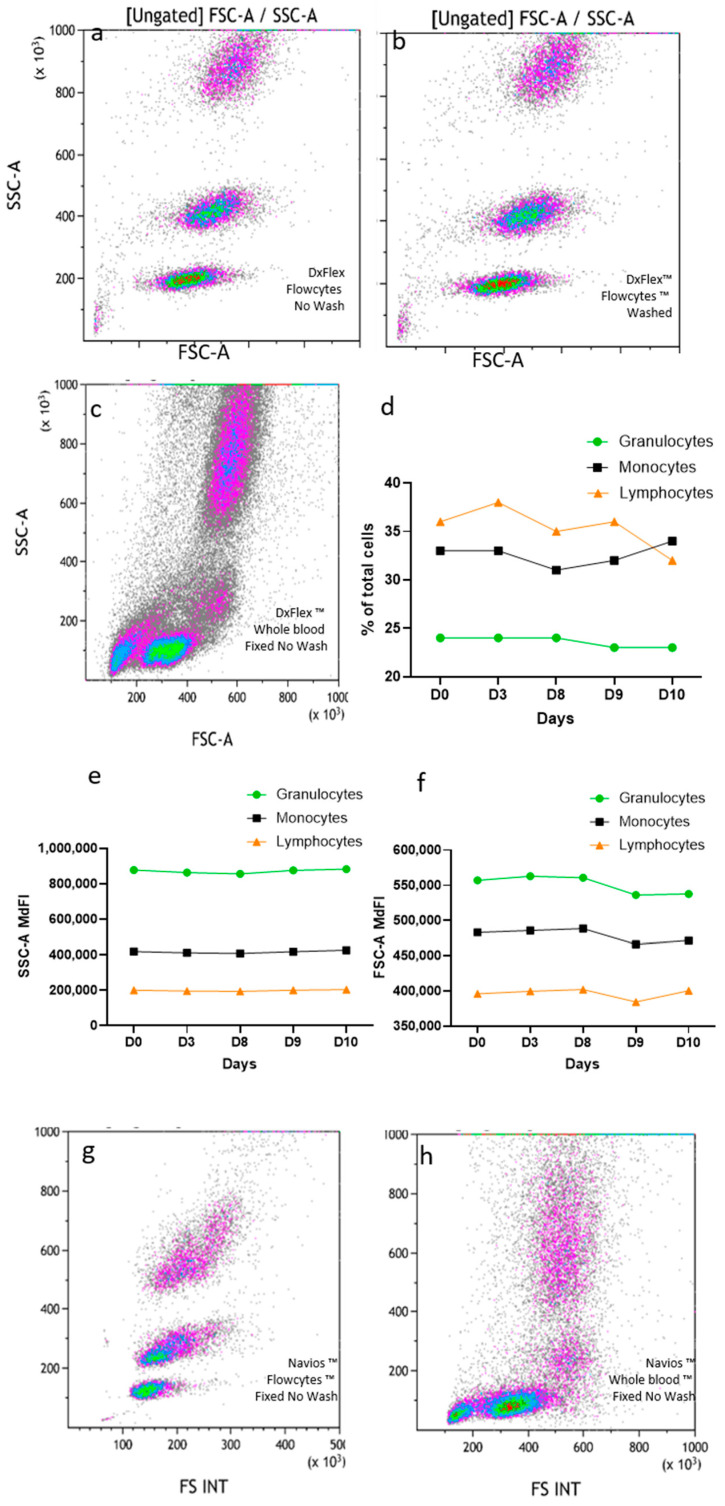
FlowCyte forward/side scatters on DxFLEX: lysis, fixation, and no washing (**a**) or with washing (**b**) compared to human blood (**c**). FlowCyte stability was checked daily for ten days, regarding subset proportions (**d**), SSC (**e**), and FSC (**f**). FlowCyte forward/side scatters on Navios (**g**) compared to human blood (representative result; (**h**)) with the lysis, fixation, no-wash protocol.

FlowCytes were stained, lysed, and fixed following the same protocol used for human blood, with the same instrument settings and the same FSC threshold. The FlowCyte forward/side scatter dot plot on DxFlex appeared to be very similar to what is usually seen with human blood, with three distinct populations of cells mimicking lymphocytes, monocytes, and granulocytes (Figure 1a,c). When the FlowCytes were not washed (using the lysis/fixation/no-wash protocol used in routine diagnosis) (Figure 1a) the dot plots looked exactly the same as when washed (as recommended by the manufacturer; Figure 1b). The three leukocyte populations could be easily gated for quantitation, and their relative proportions were different from the usual proportion values found in blood. Indeed, lymphocyte surrogates represented 35.5 + 2.0% of FlowCytes, monocytes represented 32.3 + 1.8%, and granulocytes represented 23.7 + 0.5%, while the human blood sample usually had around 19% of lymphocytes, 8% of monocytes, and 59% of granulocytes, (Figure 1c). The analysis was repeated five times on the same day, by the same technician, showing very good repeatability (Figure 1d), and daily testing over 10 days (three different technicians) showed very good stability of SSC (CV = 2.1%; Figure 1e) and FCS coordinates (CV = 1.8%; Figure 1f) over time. Using the same lysis/fixation/no-wash protocol, FlowCytes were successfully detected on Navios (Figure 1g) and displayed similar FSC/SSC properties compared to human blood (Figure 1h).

No significant differences were observed when the fixation step was removed or when the acquisition was performed at low (as recommended) or high speed. No residual FlowCytes were detected when running water-containing tubes after the FlowCyte-containing tubes, showing the absence of carryover of the sample probe or the flow cell.

### 3.2. TruCytes

**Figure 2:**
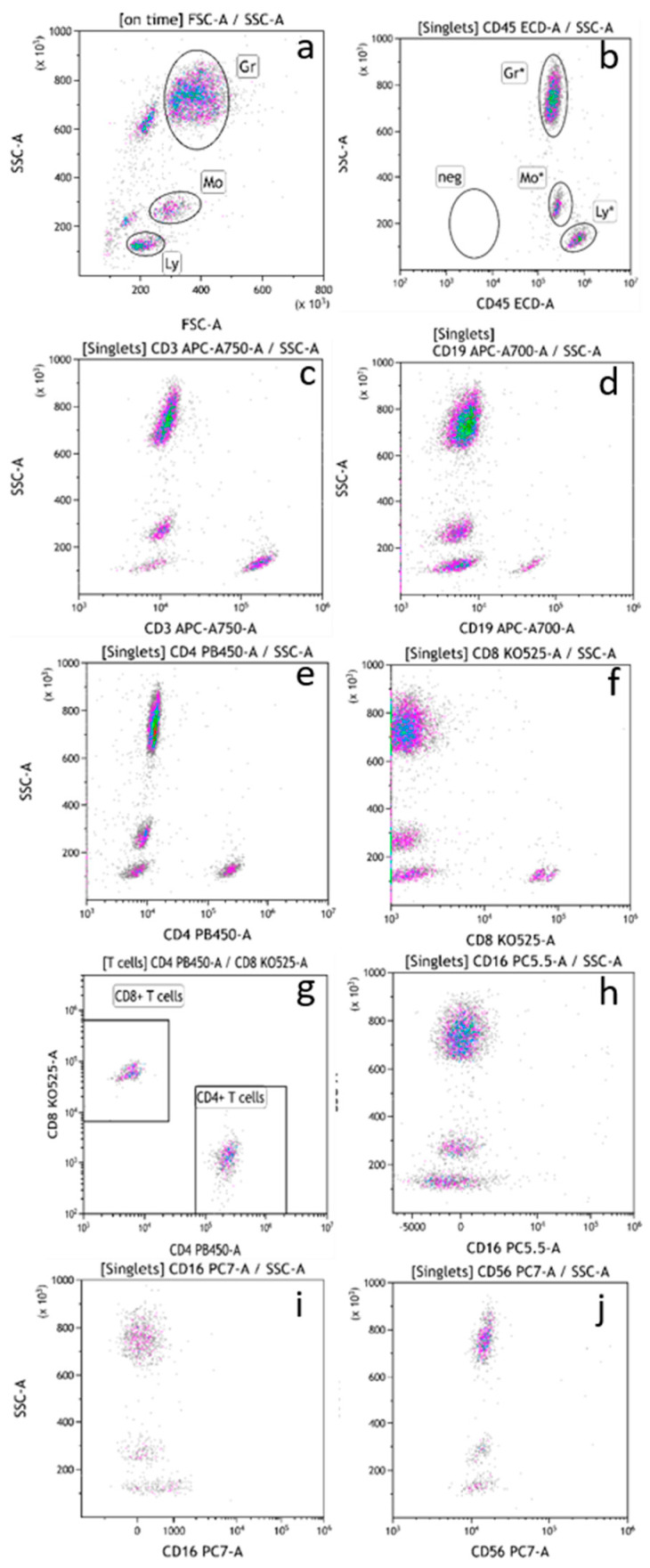
TruCytes (immunostained, fixed, no wash) forward/side scatters analyzed on DxFLEX (**a**). CD45 expression on TruCytes (**b**). CD3 (**c**), CD19 (**d**), CD4 (**e**,**g**), and CD8 (**f**,**g**) expression on TruCytes. CD16 expression on TruCytes, with a PC5.5-conjugated antibody (**h**) and with a PC7-conjugated antibody (**i**). CD56 expression on TruCytes (**j**).

TruCytes were stained, lysed, and fixed following the same protocol, with the same instrument settings and the same FSC threshold as human blood in routine analysis. TruCyte forward/side scatter dot plots appeared to be very similar to what is usually seen with human blood, with the expected three distinct populations of cells: lymphocytes, monocytes, and granulocytes (Figure 2a). The absence or presence of lysis, fixation, or washing steps did not modify the FCS/SSC values nor the speed of cell passage.

All three populations of TruCytes expressed CD45 (Figure 2b). Interestingly, CD45 median fluorescence intensity (MdFI) was higher on lymphocytes (MdFI = 1,130,979 ± 53,368; mean of five repeats) compared to monocytes (562,639 ± 44,722) and granulocytes (414,366 ± 34,680). In human blood, lymphocytes usually display higher CD45 MdFI compared to monocytes. In TruCytes, lymphocytes (18.3 ± 1.72% of all events; mean of five repeats), monocytes (10.1 ± 0.25%), and granulocytes (61.8 ± 1.04%) had similar distributions as what are usually observed in human blood [9].

CD3 (Figure 2c), CD19 (Figure 2d), CD4, and CD8 (Figure 2e–g) were detected with high signal/noise ratios despite the absence of sample washing. MdFI data are summarized in Table 2. CD3 MdFI on CD3+ TruCytes was higher (MdFI = 155,444 ± 6596; five repeats) compared to human blood T cells (MdFI = 42,148 ± 4392; seven representative patients). Similarly, CD4 MdFI was higher on CD4+ TruCytes (MdFI = 165,185 ± 41,220) compared to human blood CD4+ T cells (MdFI representative value = 65,536 ± 5050). CD8 MdFI was higher on CD8+ TruCytes (MdFI = 141,409 ± 25,942) compared to human blood CD8+ T cells (MdFI = 44,161 ± 21,837). However, CD19 MdFI was similar between CD19+ TruCytes (MdFI = 44,424 ± 7956) and human blood B cells (MdFI = 58,641 ± 19,969). Surprisingly, CD4 expression was not detected at all on monocytes (Figure 2e), while it is usually observed on monocytes from human blood. Moreover, CD16 (clone 3G8) conjugated with PE-Cy5.5 (Figure 2h) or PE-Cy7 (Figure 2i) was not detectable on TruCytes, even though granulocytes from human blood highly express CD16. Similarly, CD56 was not detectable on lymphocytes using the NCAM16.2 clone labeled with PE-Cy5.5 or the N901 clone conjugated with PE-Cy7 (Figure 2j).

**Figure 3:**
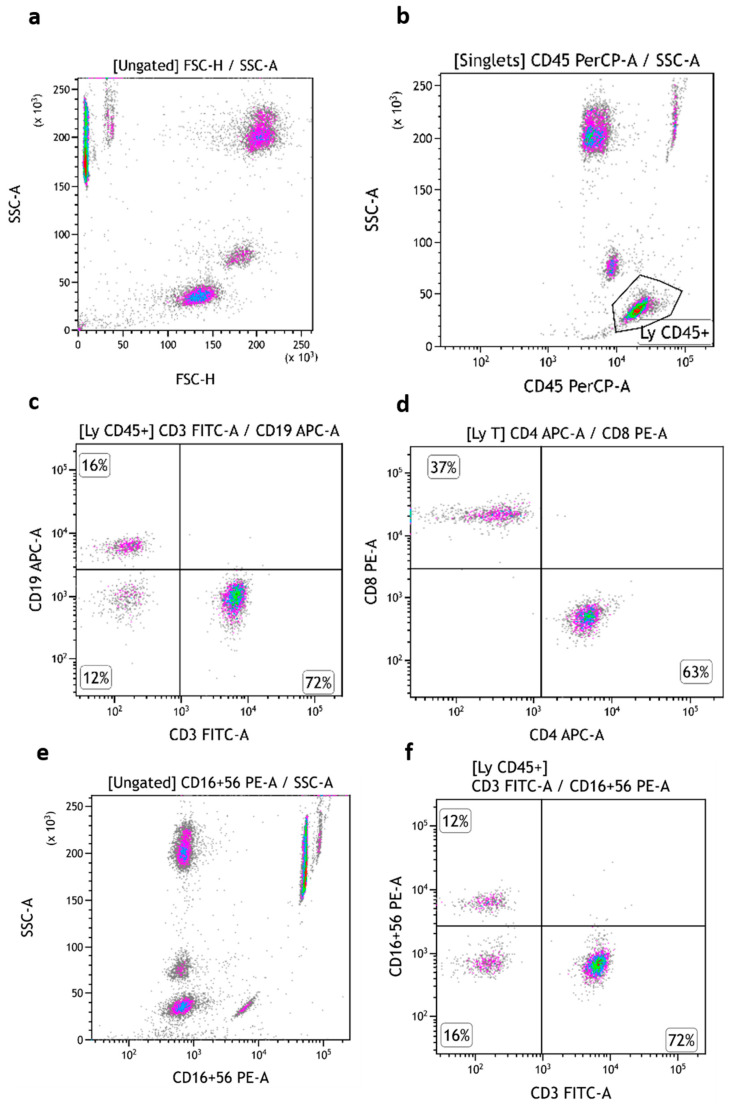
TruCytes analyzed on FACSCanto (with Trucount tubes): light scatters (**a**); CD45 (**b**), CD3/CD19 (**c**), CD4/CD8 (**d**) and CD16-CD56 (**e**,**f**). Data re-analyzed on Kaluza.

**Figure 4:**
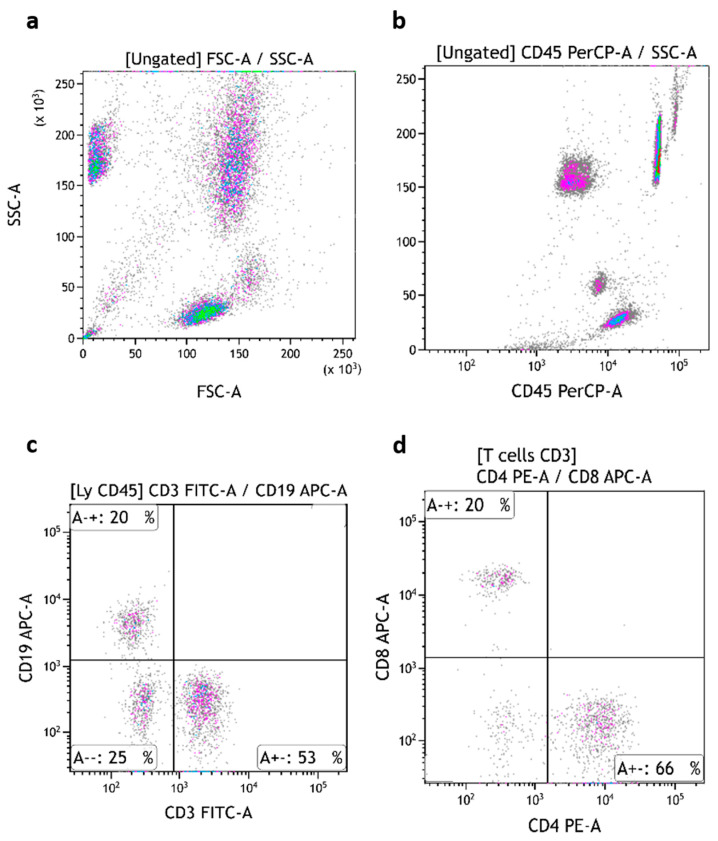
TruCytes analyzed on FACSLyric (with Trucount tubes): light scatters (**a**); CD45 (**b**), CD3/CD19 (**c**) and CD4/CD8 (**d**). Data re-analyzed on Kaluza.

**Figure 5:**
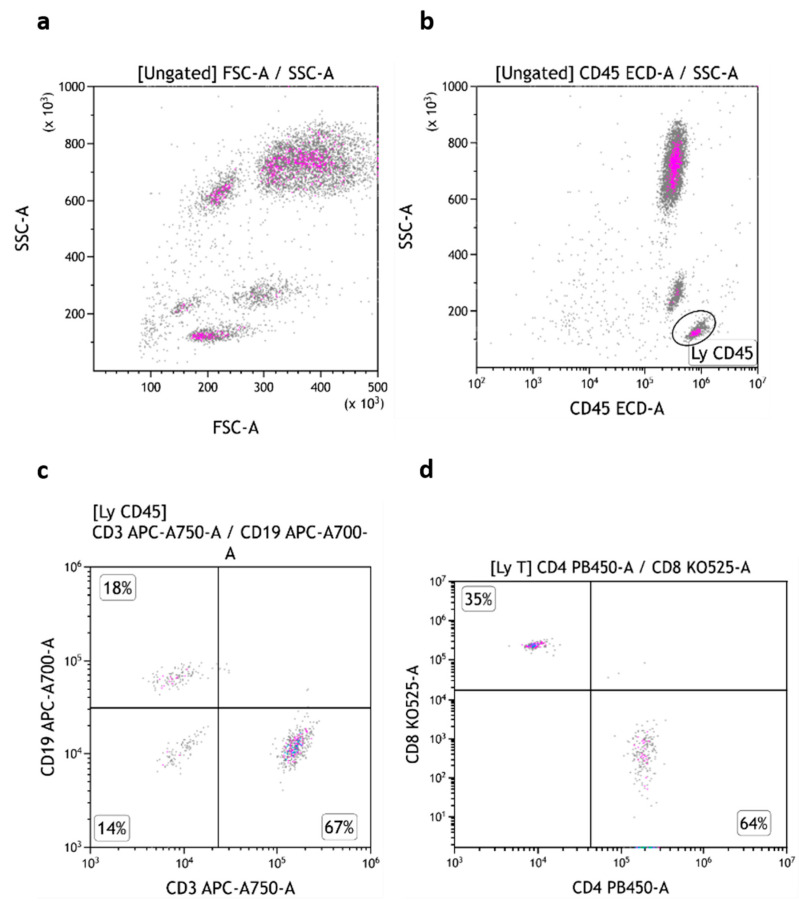
TruCytes analyzed on DxFLEX (with Flow-Count beads): light scatters (**a**); CD45 (**b**), CD3/CD19 (**c**) and CD4/CD8 (**d**). Data re-analyzed on Kaluza.

**Figure 6:**
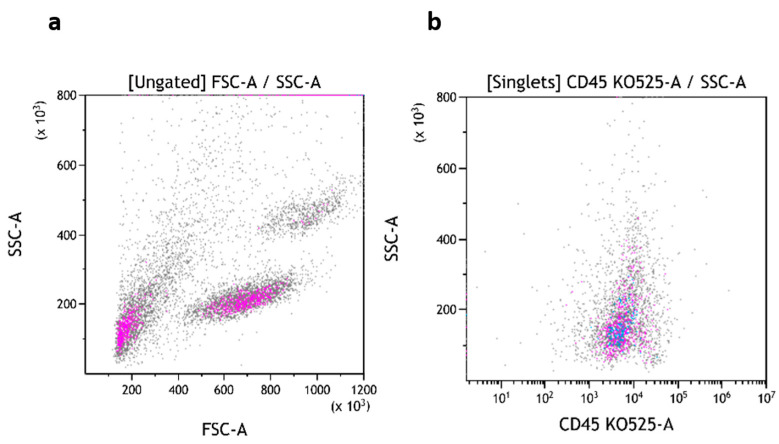
TruCytes analyzed on Navios: light scatters (**a**) and CD45 (**b**). Data re-analyzed on Kaluza.

The three subsets of TruCytes (lymphocytes, monocytes, and granulocytes) were similarly detected on FACSCanto (Figure 3a), FACSLyric (Figure 4a), and DxFLEX (Figure 5a). CD45 (Figure 3b, Figure 4b and Figure 5b), CD3, CD19 (Figure 3c, Figure 4c and Figure 5c), CD4, and CD8 (Figure 3d, Figure 4d and Figure 5d) expression was easily detected. However, CD16 and CD56 were not detectable on most systems, but CD56 was well detected on a CD3-negative lymphocyte population on a BD Biosciences system (FACSCanto, BD Multitest six-color TBNK reagent; Figure 3e,f). Notably, CD16 was not detectable on “artificial cells” mimicking neutrophils. Surprisingly, TruCyte light diffusion was poorly detectable on Navios, even after changing the forward small-angle (n, w, and w^2^) options for the detection of forward light diffusion (Figure 6a). Consequently, the three leukocyte subsets could not be identified at all, even when using CD45 (Figure 6b).

### 3.3. Labeling Quality Assessment of TruCytes (Performed on DxFlex)

**Figure 7:**
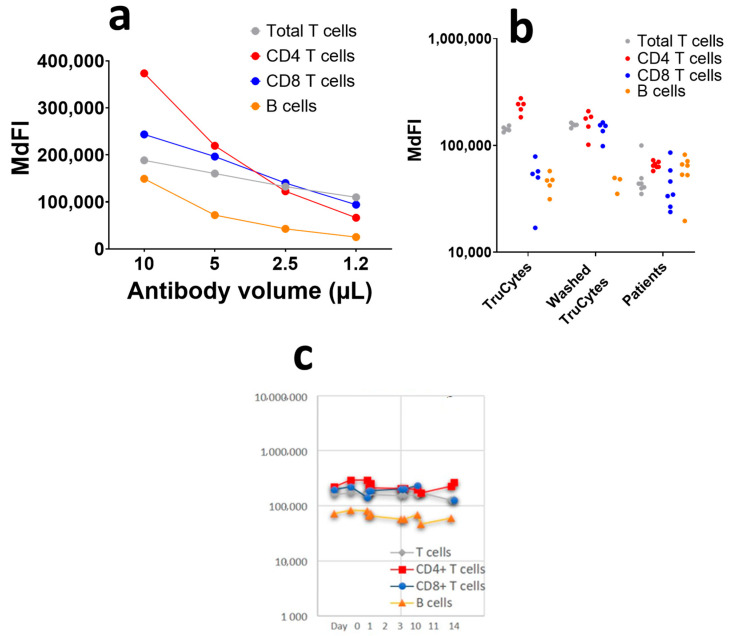
(**a**) Antibody titration on 100 µL of TruCytes on DxFLEX. (**b**) Five repeats of staining with lysis, fixation, and no washing or with washing compared to human blood. (**c**) Reproducibility of immunolabelling over 14 days using the same lot of aliquots.

The serial ½ dilutions of the antibody, starting at the recommended saturating antibody concentration (10 µL for 100 µL of whole blood), showed that the TruCyte MdFI decreased as the antibody concentrations decreased (Figure 7a). This suggests that 10 µL of antibodies for 100 µL of TruCytes was not enough to reach saturation, which was most probably due to an excess of antigen expression on Trucytes compared to human leukocytes. Despite the fact that the antibodies were not saturating, the TruCytes’ MdFI appeared to be very stable in five repeats (Figure 7b) and were higher than what was observed in seven representative patients, suggesting that the expression of all markers was higher on TruCytes compared to human lymphocytes. Lymphocyte subset distribution was similar to what is commonly observed in human blood, with 69.5 + 5.2% of T cells (5 repeated tests) and 14.1 + 4.7% of B cells. CD4+ T cells represented 64.9 ± 6.9% and CD8+ T cells represented 35.0 ± 6.9% of CD3+ T cells. No CD4/CD8 double-negative or double-positive CD3+ T cells were detected. Repeated counting resulted in coefficients of variation (CV) of 7.5% for T cells, 10.6% for CD4+ T cells, 19.7% for CD8+ T cells and as high as 33.1% for B cells. The CV significantly increased when the size of the cell population was lower. Daily analyses performed over a period of 14 days, using two vials of the same lot, indicated high reproducibility (Figure 7c), with CV values of 6.13% for T cells, 5.8% for CD4+ T cells, 7.7% for CD8+ T cells, and 15.1% for B cells.

### 3.4. Comparative Inter-Laboratory Analyses of TruCytes

**Figure 8:**
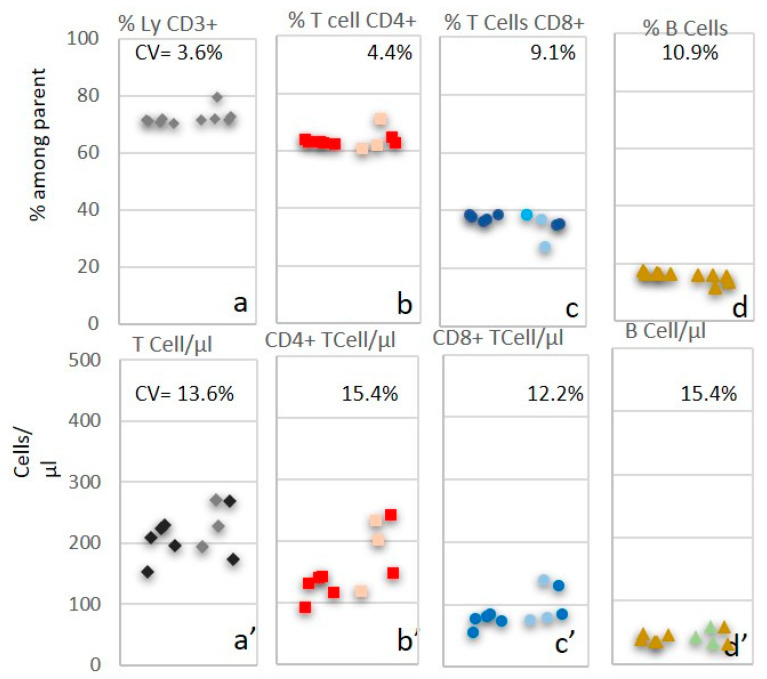
TruCyte aliquot analyses from 4 different laboratories in France performed on 8 cytometers, using the same local protocol and instrument settings for TBNK immunophenotyping and counting. Results with Navios are in light colors. First four results (left) were from Trucount tubes (BD Biosciences), while results in the right columns were obtained using Flow-Count (Beckman-Coulter) beads. Relative counts of CV were 3.6% for T cells (**a**), 4.4% for CD4+ T cells (**b**), 9.1% for CD8+ T cells (**c**), and 10.9% for B cells (**d**). Absolute counts of CV were 13.6% for T cells (**a’**), 15.4% for CD4+ T cells (**b’**), 12.2% for CD8+ T cells (**c’**), and 15.4% for B cells (**d’**).

In order to test whether TruCytes could be used as EQC, a vial of the same lot was sent to different clinical laboratories in France. Each laboratory was asked to conduct the analysis of TruCytes using the same cytometer settings and sample preparation protocol it routinely uses for T, B, and NK subset immunophenotyping. Results from laboratories using Navios confirmed a defect in the detection of TruCytes on this instrument; we made the same observation locally and excluded it from the analysis. Results originated from four laboratories using eight different cytometers (Figure 8) displayed CV values of 3.6% for T cells, 4.4% for CD4+ T cells, 9.1% for CD8+ T cells, and 10.9% for B cells. Absolute counting was also performed using either Trucount tubes or Flow-Count beads, resulting in CV values of 13.6% for T cells, 15.4% for CD4+ T cells, 12.2% for CD8+ T cells, and 15.4% for B cells.

## 4. Discussion

Our results highlight the innovative potential of artificial cell mimics developed by Slingshot Biosciences. While other microparticles—such as plastic beads (e.g., CBA fromBD Biosciences) or antigen-coated gel polymers (e.g., FlowPRA beads for detecting alloreactive antibodies in transplantation)—have been widely used in flow cytometry [17,18,19,20], FlowCytes and TruCytes are uniquely designed to mimic both the physical and optical properties of human leukocytes. Although their chemical and structural details remain proprietary, our study demonstrates that these mimics exhibit forward and side scatter (FSC/SSC) profiles remarkably similar to those of human leukocytes on most CE-IVD cytometers.

The use of routine lysis/fixation/no-wash protocols, which differ from the manufacturer’s recommendations, did not alter the FSC/SSC dot plots of FlowCytes and TruCytes. This robustness is essential for their potential use as quality controls as they must be processed and analyzed in the same manner as patient samples.

However, TruCytes exhibited significantly altered light scattering on Navios systems, suggesting differences in diffraction indices. This limitation likely extends to Gallios and FC500 systems, which were not tested here but share similar optical configurations. The alteration persisted across all FSC small-angle options available on Navios (N, W, and w^2^) and may be related to the shorter distance of the FSC detector on Navios compared to FACSCanto and DxFlex. This phenomenon is not unique to TruCytes; beads from one manufacturer are often incompatible with cytometers from other companies, highlighting a broader challenge in cross-platform standardization.

TruCytes demonstrated robust immunostaining of CD45, CD3, CD4, CD8, and CD19, even when processed with lysing and fixative solutions without washing, and with monoclonal antibodies from various providers. The differential expression of CD45 on lymphocytes, monocytes, and granulocytes mirrored that observed in fresh human leukocytes [4]. The fluorescence intensities and subset distributions of CD3, CD4, CD8, and CD19 were comparable to those in human blood, although CD4 was not expressed on monocytes—a departure from human leukocyte profiles. The proportions of lymphocyte subsets were similar to those in human blood, and the absence of double-negative or double-positive T cells is not a significant limitation for basic clinical interpretation or quality assurance. These rare T cell subsets, which express CD4 and CD8 at asymmetrical levels or neither marker, are thought to play specialized roles in immune defense, particularly in mucosal immunity and chronic infections such as cytomegalovirus, and they are of interest in the detection of primary immunodeficiencies [7,8].

However, the inability to detect NK cells using monoclonal antibodies other than those validated by the manufacturer is a critical drawback. NK cell mimics are likely present in TruCytes, as the combined percentage of T and B cells did not exceed 85% of total lymphocytes. This suggests that the CD56 epitopes on TruCytes are not recognized by commonly used antibody clones, limiting their utility for NK cell validation. Additionally, CD16 was undetectable on granulocyte and monocyte mimics despite its significant expression on human cells. These limitations reduce the value of TruCytes for comprehensive lymphocyte subset enumeration and blood count validation.

The use of counting beads allowed for the determination of cell concentrations with good precision, a critical requirement for routine diagnostics. However, the low concentration of cell mimics in TruCyte vials—significantly below typical human leukocyte concentrations—poses a challenge for quality control applications as QCs should ideally span the normal range of patient sample concentrations. The reconstitution of lyophilized TruCytes with distilled water introduces additional variability, although our experience showed good stability of reconstituted samples for up to 10 days, exceeding the manufacturer’s 7-day guarantee. The risk of pipetting errors during reconstitution increases with the number of aliquots, but our data indicate that TruCytes can serve as internal QCs for relative and absolute counts of T and B cells.

In inter-laboratory comparisons, TruCytes yielded highly similar relative counts across different cytometers and protocols, with coefficients of variation (CV) comparable to those reported in international quality assessment programs such as UK NEQAS [21]. This suggests their potential as external QCs. However, absolute counting variability was higher, likely due to the known poorer reproducibility of absolute counts at low concentrations [22] and the slight overestimation typically observed with Beckman Coulter cytometers and Flow-Count beads compared to BD Biosciences systems and Trucount tubes [21].

Thus, FlowCytes and TruCytes appear highly suitable for use as QCs in routine laboratories, even when using protocols that deviate from the manufacturer’s instructions. However, several limitations must be addressed: the lack of recognition of CD56 by commonly used antibody clones, the absence of CD16 expression on granulocyte and monocyte mimics, poor detectability on Navios cytometers, and the low concentration of cell mimics compared to human blood. Relevant IQC or EQC must be validated across the normal range of concentrations found in patient samples, ideally at 2–3 different levels throughout the analytical range. Additional limitations include the requirement for dry ice shipping and the critical reconstitution step, both of which introduce logistical and technical challenges.

## 5. Conclusions

FlowCytes and TruCytes effectively mimic the light scattering properties of fresh human leukocytes on most commonly used CE-IVD cytometers, making them promising tools for quality control in T and B cell immunophenotyping. Their compatibility with routine lysis/fixation/no-wash protocols and robust immunostaining for key markers are notable advantages. However, to enhance their utility, several limitations must be addressed: the lack of recognition of CD56 by commonly used antibody clones, the absence of CD16 expression on granulocyte and monocyte mimics, poor detectability on Navios cytometers, and the low concentration of cell mimics compared to human blood. With targeted improvements, TruCytes could become a highly convenient and reliable tool for the validation of T, B, and NK cell immunophenotyping in routine laboratories.

## Figures and Tables

**Table 1:** Different clones used for immunolabeling according to the IVD-CE-certified systems that were tested.

Markers	Slingshot Biosciences Recommended	BD Biosciences Limoges	Beckman Coulter	Beckman Coulter
CD45	2D1	2D1	J33	B3821F4A
CD16	B73.1	B73.1	3G8	3G8
CD56	NCAM16.2	NCAM16.2	N901	N901/NKH-1
CD19	SJ25C1	SJ25C1	J3-119	J3-119
CD3	SK7	SK7	UCHT1	UCHT1
CD4	SK3	SK3	13B8.2	SFCI12T4D11
CD8	SK1	SK1	B9.11	SFCI21Thy2D3

**Table 2:** Median fluorescence intensities according to cell type.

		Median Fluorescence Intensity (MdFI)			
Cell type		CD3	CD4	CD8	CD19
	CD3+ TruCytes	155,444			
	Human CD3+ T cells	42,148			
	CD4+ TruCytes		165,185		
	Human CD4+ T cells		65,536		
	CD8+ TruCytes			141,409	
	Human CD8+ T cells			44,161	
	CD19+ TruCytes				44,424
	Human CD19+ B cells				58,641

## Data Availability

The original contributions presented in the study are included in the article. Further inquiries can be directed to the corresponding author.

## Abbreviations

EQC	External Quality Control
IQC	Internal Quality Control
QA	Quality Assurance
IVDR	In Vitro Diagnostic Regulation 2017/746
MdFI	Median Fluorescence Intensity
EDTA	Ethylene Diamine Tetra Acetic
FCM	Flow Cytometry
BD	Beckton Dickinson
BEC	Beckman Coulters
FSC	Forward Scatter
SSC	Side Scatter
NS	Not Significant
FITC	Fluorescein Isothiocyanate
PE	Phycoerythrin
Cy	Cyanin
APC	Allophycocyanin
PBS	Phosphate-Buffered Saline
